# Sjögren’s Syndrome Treatments in the Microbiome Era

**DOI:** 10.20900/agmr20230004

**Published:** 2023-05-06

**Authors:** Christian Furlan Freguia, David W. Pascual, Gary R. Fanger

**Affiliations:** 1 Rise Therapeutics, 1405 Research Blvd., Rockville, MD 20850, USA; 2 Department of Infectious Diseases & Immunology, College of Veterinary Medicine, University of Florida, P.O. Box 110880, Gainesville, FL 32611, USA

**Keywords:** Sjögren’s syndrome, microbiome

## Abstract

Sjögren’s syndrome (SS) is a chronic autoimmune disease characterized by inflammatory cell infiltration of the salivary and lacrimal glands, resulting in acinar epithelial cell atrophy, cell death, and loss of exocrine function. At least half of SS patients develop extraglandular inflammatory disease and have a wide range of systemic clinical manifestations that can affect any organ system, including connective tissues. As many as 3.1 million people in the U.S. suffer from SS, a disease that causes severe impairment. Women are nine times more likely than men to be affected by this condition. Unfortunately, there is currently no effective treatment for SS, and the available options only provide partial relief. Treatment involves using replacement therapies such as artificial saliva and eye lubricants, or immunosuppressive agents that have limited efficacy. The medical community recognizes that there is a significant need for more effective treatments for SS. Increasing evidence demonstrates the links between the dysfunction of the human microbial community and the onset and development of many human diseases, signifying the potential use of microorganisms as an alternative strategy to conquer these issues. The role of the microbiome in controlling immune function of the human host in the context of autoimmune diseases like SS is now becoming better understood and may help to enable new drug development strategies. Natural probiotics and synthetic biology applications hold promise for novel treatment approaches to solve the encryption of many complex and multifactorial immune disorders, like SS.

## THE ROLE OF THE IMMUNE RESPONSE IN SS

Sjögren syndrome (SS) is an autoimmune disease characterized by ocular and oral dryness resulting from lacrimal and salivary gland dysfunction [1,2]. The dryness also affects other mucosal surfaces such as the airways, digestive tract, and vagina, resulting in the clinical picture of “sicca syndrome” or “sicca complex” [3,4]. SS can affect the secretory function specifically, defined as primary SS, or the disease can involve virtually any organ system, leading to extremely pleomorphic clinical manifestations associated with systemic autoimmune rheumatic diseases (secondary SS) [5]. This classification is somewhat historical as more recently the disease is described as either a standalone disorder or associated with other autoimmune complications to account for the fact that patients can develop other autoimmune diseases subsequent to SS [6,7]. The severity of SS is quite broad, spanning mild glandular dryness and constitutional symptoms to severe glandular involvement and a variety of extraglandular manifestations and systemic autoimmune features. This creates challenges in establishing the diagnosis, differentiating the condition from other systemic autoimmune disorders or causes of salivary gland enlargement [8,9]. There is no single diagnostic test for SS and more sophisticated assays are needed to catch patients early on during disease onset and start treatments sooner.

SS mainly affects middle-aged women, with an average female to male ratio 9:1 [3], with an incidence of 6.92 per 100,000 people/year, and a prevalence of 60.82 per 100,000 people [10,11]. Because of the heterogeneity of the clinical manifestations, in many cases, SS can go undiagnosed. Patients may start to experience symptoms well before a formal diagnosis is made. Aspects of disease including autoantibodies and/or laboratory features (such as hypergammaglobulinemia and lymphopenia) can present years before diagnosis [12]. Around 60% of people with SS develop the disease as a secondary condition to an existing underlying autoimmune disorder such as rheumatoid arthritis (RA), systemic lupus erythematosus (SLE), or multiple sclerosis. [13]. Currently, there is no cure for SS, and the treatment options are extremely limited for a disease that significantly alters the quality of life [14]. Dryness in the mouth can significantly impact essential everyday activities including eating, speaking, and sleeping. When the saliva production decreases, the antibacterial properties of the oral cavity decrease, increasing the risk of infections, tooth decay, and periodontal disease [15]. Patient suffering from SS also commonly experience complications of the eye such as itching, soreness, grittiness, and dryness, even though the eyes appear normal. This is due to a decrease in tear production, which can cause chronic irritation and damage to the corneal and bulbar conjunctival epithelium, known as keratoconjunctivitis sicca. In addition, SS increases the risk of mortality by 50% [16].

Central to the pathophysiology of SS is a chronic immune system stimulation [17]. In primary SS, mononuclear inflammatory infiltrates and IgG plasma cells that infiltrate the salivary and lacrimal glands contribute to damage and ultimately irreversible destruction of the glandular tissue [18]. While the processes that underlie the humoral and cellular autoimmune reactions observed in patients with SS are not known, B cells play a central role in the immunopathogenesis and exhibit signs of hyperactivity [19]. Hyperactivity of B cells results in secretion of autoantibodies and production of various cytokines, including type I IFN, BAFF, IL-6, and IL-21 [20]. T lymphocytes are also involved in the disease etiopathogenesis [21]. Helper T cell subsets converge at different stages of the disease and contribute to pro-inflammatory cytokine secretion in target tissues. Accumulation of immunomodulatory T cell-derived factors, such as IL-17, IFN-γ, or IL-21, not only preserve the inflammatory environment, but also favor strong B cell and T follicular helper (Tfh) cells activation [22]. Lymphocytic infiltration of the salivary and lacrimal glands is characterized by a presence of CD4^+^ T cells at the early stages of the disease, followed by B cell accumulation at later stages [23].

The etiology of SS is unknown and may include exposure to specific environmental factors in genetically susceptible individuals [24,25]. Among environmental factors, the gut microbiome is emerging as a potential contributor to the disease etiopathogenesis.

## THE IMMUNOMODULATORY ROLE OF THE GUT MICROBIOME

Over the last ten years, there has been a significant increase in research related to the microbiome, revealing an important relationship between gut bacteria and their human hosts. The gut microbiota is now recognized as a critical factor in regulating overall host health [26]. The gut bacteria have been acknowledged for their role in aiding digestion, producing essential nutrients such as vitamins, and protecting the body from harmful pathogens. Microbial profiles correlate to immune competence, metabolic activities, neurologic and cardiovascular functions, and cancer risk. Moreover, there is a connection between the disturbance of the natural microbiota and various human ailments. Deviations in the gut microbiota have been linked to numerous health conditions, such as obesity, type 2 diabetes, hepatic steatosis, intestinal bowel diseases (IBDs), and multiple types of cancer [27].

The central role of the gut microbiome in health and disease stems from the complex interplay of the microbiome with the host immune system. The gut microbiome plays a critical role in training and development of the host’s immune system. Microbiota and innate immunity are engaged in an extensive bidirectional communication that is the results of specific signaling molecules produced by the host as well as the gut bacteria that culminate in the activation of monocytes, macrophages, and Innate lymphoid cells (ILCs) that lie the gut endothelial barrier [28–30]. Particularly important is the relationship between the microbiota and tissue-resident dendritic cells (DCs), which are central to the development of immune memory and tolerance [31,32]. Recent studies have revealed mechanisms that regulate the mutualistic relationship between the microbiome and the adaptive immune system, in addition to their impact on innate immune function. Crosstalk between gut microbes and B cells, via IgA production, facilitates the expansion of Foxp3^+^ regulatory T cells (Tregs) [33,34] and the interaction with colonic regulatory CD4^+^ T cells supports gut homeostasis [35,36]. The microbiome regulation of adoptive T cell responses involves CD8^+^ (cytotoxic) T cells, which works in the elimination of intracellular pathogens and cancer cells. An important microbiome-host interaction is between gut bacteria and Tfh cells, which are specialized to assist B cells, and are implicated in maintenance of microbiota homeostasis [37–39].

Given the complex and wide interaction between the gut microbes and the host immune system, imbalances in microbiota community can contribute to the pathogenesis of a multitude of immune-mediated disorders. Environmental factors such as changes in diet, geography, or the use of antibiotics can disrupt the gut microbiome, impair host-microbiome interfaces, or alter the immune function leading to an abnormal immune response. The interactions between the microbiome and the immune system are associated with several gastrointestinal diseases such as IBD and celiac disease, as well as non-gastrointestinal disorders like rheumatoid arthritis, metabolic syndrome, CNS disorders, and cancer [40–45].

## IMPLICATIONS OF GUT, ORAL, AND OCULAR MICROBIOME DYSBIOSIS IN SS

**Figure 1.**
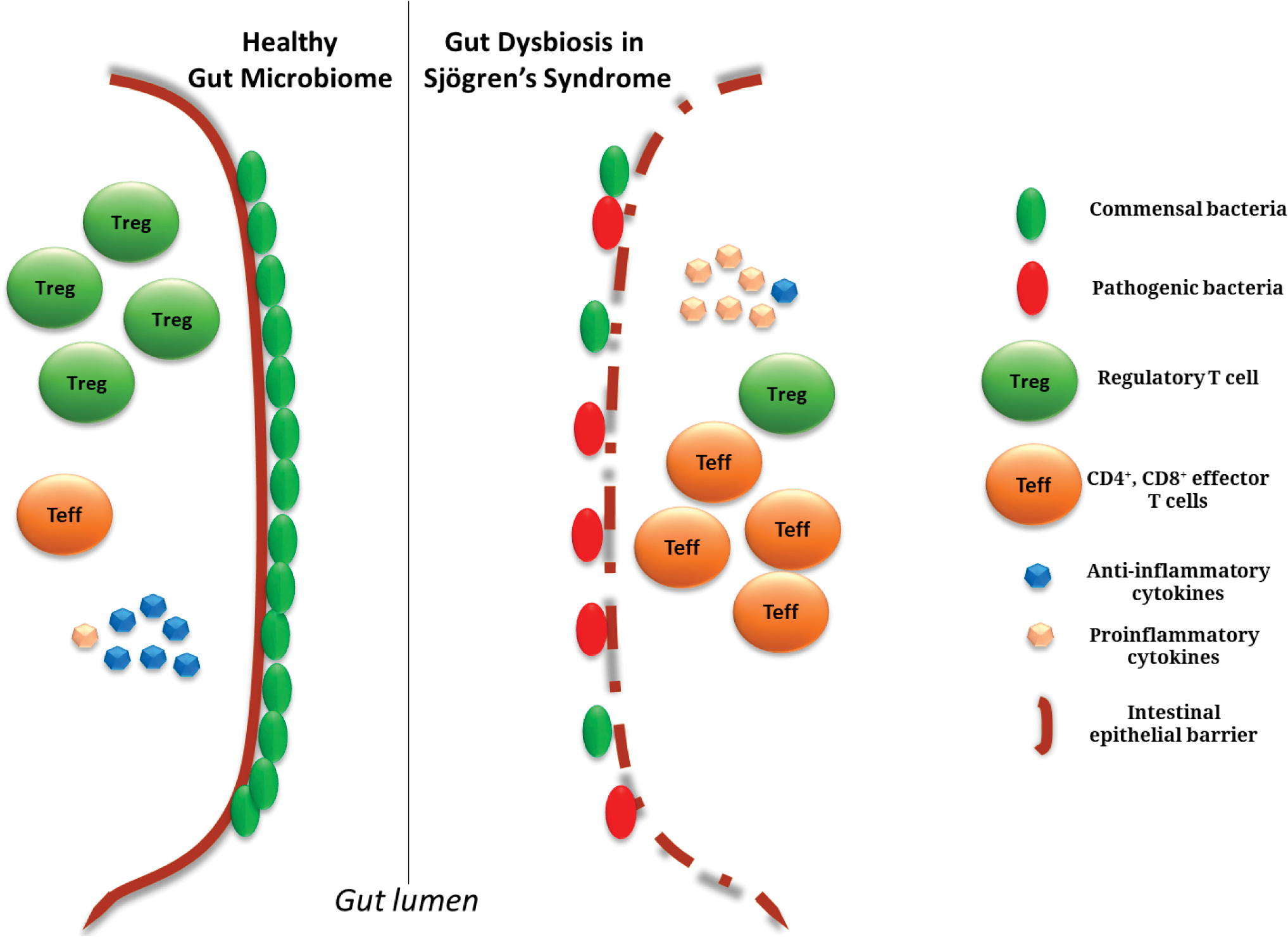
Gut microbiome changes in SS patients. SS patients present an altered gut microbiome characterized by (1) a reduction of bacteria richness and appearance of pathobionts; (2) expansion of effector (Teff), CD4^+^ and CD8^+^ T cells to the detriment of regulatory T cells (Tregs); (3) an increase in proinflammatory cytokines, replacing the anti-inflammatory signals; and (4) disruption of the intestinal epithelial cell integrity.

As microbiota diversity is essential to maintain the stability and homeostasis of the gut, microbiome alterations can result in imbalance of the immune response leading to chronic disorders. Growing evidence has shown that SS is also characterized by microbial perturbations [46–48] (Figure 1). Several studies have demonstrated that SS patients present significant gut dysbiosis. In these studies, gut dysbiosis is correlated with disease severity [47,49–51]. As an example, in a study of 42 SS patients, severe dysbiosis was more prevalent in SS patients and more severe dysbiosis correlated with higher disease activity as evaluated by the ESSDAI total score and the ClinESSDAI total score, as well as higher levels of fecal calprotectin [51]. The gut microbiome of SS patients is positively associated with the richness of Enterobacter, while negatively associated with the abundance of *Lachnospira*, *Roseburia*, *Bifidobacterium*, *Ruminococcus*, *Blautia*, and *Roseburia* [47,50,52]. Dysbiosis observed in autoimmune disease such as SS is associated with systemic inflammation [53,54]. The levels of certain proinflammatory factors such as IL-6, IL-12, IL-17, and TNF-α are associated with changes in the gut microbiome. Interestingly, germ-free mice colonized with human intestinal microbiota from SS patients showed a reduced frequency of CD4^+^ FOXP3^+^ Tregs further strengthening the link between gut dysbiosis and SS [49]. The gut microbiome dysbiosis can modulate systemic inflammation which in turn can diminish beneficial gut bacteria [50,55]. As we are gaining more information on the alterations of the gut microbiome in SS patients, causality studies are still needed to better understand the disease etiopathogenesis. Moreover, microbiota alterations do not only refer to changes in bacteria diversity and richness, but also perturbation in their metabolites, which can also be implicated in SS disease onset and progression.

Microbiota diversity is essential to maintain the stability and efficiency of the body. In addition to the gut, microbial dysbiosis is observed in SS patients in the oral cavity and eyes, and they may all contribute to the development of SS [48]. The relationship of the so-called gut-ocular-oral axis has been addressed by several studies [46,47,56,57]. In both animal models and human studies, dysbiosis of the gut microbiota was shown to partly correlate with changes in the oral and ocular microbiome. Therefore, it is plausible that there is a link between oral/ocular and gut bacteria but the related mechanism is still unknown. Understanding the causality relationship could be challenging since decreased salivary and lacrimal flows severely impact the bacteria species inhabiting those tissues, hence changes in the ocular/oral microbiome could be more a consequence rather than a cause of the disease.

Alterations of the gut microbiome in SS patients can also affect the brain via the gut-brain axis. Depression is quite prevalent in SS patients and recent evidence suggest a crucial role of the gut microbiome, as also recently confirmed in animal models [58–60]. In addition to depression, fatigue, fibromyalgia, and lung manifestations are also common in SS patients [61–63]. These complications are associated with an impaired gut microbiome as well [64–66].

While it is clear that microbiome alterations have an effect on the pathogenesis of SS and potentially the onset of secondary complications, the causality is still incompletely understood.

## LEVERAGING THE GUT MICROBIOME-HOST IMMUNE SYSTEM TO IMPROVE SS

Based upon our understanding of the role of dysbiosis in the onset and progression of autoimmunity, a provocative new strategy to intervene in diseases such as SS is to alter the gut microbiome to modulate the host immune response. Thus far, only a few studies have addressed microbiome interventions to modify the disease severity of SS (Table 1).

One potential intervention is fecal microbiota transplantation (FMT), which refers to a method by which donor gut microbiota is transferred into the digestive tract of the recipient, aiming to restore gut microbial imbalance [70]. FMT has been used as a core therapy for treating dysbiosis-related diseases as indicated by more than 700 studies in 85 different diseases [71], which culminated with the approval of FMT as treatment for recurrent *Clostridioides difficile* infection (CDI). There is only one FMT study reported in individuals with immune-mediated dry eye (DE) symptoms, meeting criteria for SS [72]. In this open-label, nonrandomized clinical trial, 10 individuals received 2 FMTs from a single healthy donor delivered via enema, 1 week apart. The study confirmed the safety of FMT and the potential beneficial impact as half of the subjects reported improved DE symptoms, despite no donor-bacteria engraftment observed in the gut microbiota of recipients. More FMT studies carried out in a randomized, placebo-control manner are needed to confirm whether FMT could be a strategy to ameliorate SS.

Another intervention consists in using prebiotics and probiotics, which are now at the fore center of a new wave of medications aimed at reshaping the host microbiome. Probiotics are live microorganisms which confer a health benefit to the host [73]. Prebiotics are oligosaccharides that are fermentable and non-digestible, which may modify the composition and/or function of the gut microbiota [74,75]. There is now great interest in the therapeutic potential of probiotics and prebiotics-based strategies for a range of autoimmune disorders, which has been corroborated in several studies [73,76–78]. Probiotics play a role in regulating the host innate and adaptive immune responses by modulating the functions of dendritic cells, macrophages, and T and B lymphocytes [79,80]. The immune response modulation by probiotics is strain specific and can results in shifting the T_H_1/T_H_2 balance with upregulation of certain cytokines and enhancement of the natural killer cells, as showed in human studies [81]. An important focus has been on the role of Tregs, which play a crucial role in maintaining immune homeostasis, and their levels are reduced in patients with autoimmune disorders, including SS [67,68,82]. Animal models have shown that certain probiotic strains can induce Treg expansion and downregulate effector T cells and proinflammatory cytokines [69,78,83,84]. Probiotics can stimulate regulatory dendritic cells expressing IL-10, TGF-β, COX-2, and indoleamine 2,3-dioxygenase, which in turn promote the generation of CD4^+^Foxp3^+^ Tregs [83]. The therapeutic potential of probiotics and prebiotics approaches regarding autoimmune diseases would represent a more natural way to master the autoimmune response and avoid the typical side effects of immunosuppressive drugs.

The scientific literature is replete with animal and human studies assessing the efficacy of probiotics to treat autoimmune disorders. Probiotic supplementation is linked to improved disease activity and lower inflammatory parameters in patients with rheumatoid arthritis (RA), which is a common comorbidity with secondary SS [85–87]. In a very encouraging study, 8-week probiotic supplementation in RA patients significantly improved RA clinical and metabolic status [85]. In the context of type 1 diabetes (T1D), probiotic supplementation leads to a decreased risk of islet β-cell autoimmunity [88–90], and it has been associated with a better glycemic control, increased synthesis of GLP-1 (beneficial insulinotropic gut hormone), and reduced TLR4 signaling (an inflammatory signaling) [91–93] in both adults and children [94]. Preliminary evidence shows the potential role of probiotics in ameliorating multiple sclerosis (MS), and MS patients receiving probiotics revealed significant beneficial effects of probiotic supplementation on their mental health [95,96].

A number of studies assessed the beneficial potential of probiotics in ameliorating dry eye severity, one of the most common symptoms associated with SS. In an experimental animal model of SS, probiotic therapy with a 5-strain composition of *Lactobacillus casei*, *Lactobacillus acidophilus*, *Lactobacillus reuteri*, *Bifidobacterium bifidum*, and *Streptococcus thermophilus* alleviated ocular surface disease by lowering uveitis scores and improving tear secretion [47]. In a similar preclinical model, administration of *Lactobacillus plantarum* and *Bifidobacterium bifidum* modulated the expression of proinflammatory cytokines such as TNF-α and anti-inflammatory cytokines such as IL-10 and gut microbiota composition to improve the disease severity [97]. In yet another preclinical study, administration of the same 5 commensal strains (*Lactobacillus casei*, *Lactobacillus acidophilus*, *Lactobacillus reuteri*, *Bifidobacterium bifidum*, and *Streptococcus thermophilus*) in a SS mouse model significantly increased tear secretion after 3 weeks treatment by modulating the host immune response [98].

These preclinical findings have prompted studies to address translatability of probiotic treatment strategies in humans. Participants with dry eyes receiving probiotic and prebiotic supplements for 4 months had the average Ocular Surface Disease Index score significantly improved compared to controls, providing evidence that prebiotics and probiotics might be effective in the management of dry eye disease [99]. Patients with SS are at a higher risk to develop oral candidiasis than the general population. In a clinical study, 32 SS patients were randomly allocated into two groups receiving either probiotic or placebo capsules twice a day for 5 weeks. The strains included in the probiotic capsule for this study were *Lactobacillus acidophilus*, *Lactobacillus bulgaricus*, *Streptococcus thermophilus* and *Bifidobacterium bifidum*. In the probiotic group, there was a statistically significant reduction of the candidal load from baseline to the 5th week respectively, but no significant changes were observed in the placebo group [100]. This result was not confirmed in another clinical study [101], suggesting that, while probiotics hold promise for treating autoimmune disease, their beneficial effects are still incompletely understood, and more studies are needed, especially for SS. Future research needs to better elucidate the mechanisms of probiotic function, address the person-to-person variability, and define novel biomarkers that can be used to assess efficacy and safety of probiotics in humans.

As we move forward in harnessing the therapeutic potential of the microbiome, rather than using prebiotics and probiotics, novel synthetic biology strategies could be better deployed to develop autoimmune therapies that specifically target the pathways that gut bacteria engage with the host immune cells.

## SYNTHETIC BIOLOGY FOR MICROBIOME ENGINEERING

Human gut commensal bacteria have evolved to occupy their niches by modulating the host immune responses; that is, bacteria can exert either anti-inflammatory or proinflammatory pathways to achieve a symbiotic relationship with their host. Therefore, when we use bacteria “as is”, their beneficial contribution could be masked by their propensity to engage a balanced ‘not too hot and not too cold’ Goldilocks scenario. This was first shown in vitro using *Lactobacillus plantarum*, using knock-out mutants to identify specific gene loci that modulate the immune response of dendritic cells [102], and then elegantly demonstrated in vivo by Lightfoot et al. where only after removing the *Lactobacillus* (*L.*) *acidophilus* proinflammatory signals, the therapeutic effects *L. acidophilus* strain became evident [103]. In this latter study, deletion of the immune activating cues surface layer protein (Slp) B, SlpX, and LTX, enabled enrichment of the anti-inflammatory signal mediated by SlpA to predominate, effectively improving the therapeutic activity of *L. acidophilus* to enable the synthetically engineered strain of *L. acidophilus* to treat inflammatory bowel disease in murine animal models [103].

Recent advances in synthetic biology now allow the precise manipulation of bacteria for diverse functional purposes [104]. Importantly, now that it is possible to dissect the specific signals of bacteria synthetic biology can specifically overexpress those proteins in generally recognized as safe probiotics in the context of developing functionally-directed probiotics [105]. Engineered bacteria can be designed to influence host biology in a very exquisite and directed manner, enabling precision targeted microbiome drug development. In this regard, the safe probiotic becomes the chassis to deliver the therapeutic proteins in vivo.

Among probiotics, *Lactococcus lactis* has attracted significant attention in the engineered biotherapeutic arena [106]. Initial studies with the *L. lactis* line engineered to deliver immune regulatory molecules were carried out in the context of treating colitis (Table 2). A handful of Phase 1 and Phase 2 clinical studies have been carried out using *L. lactis* engineered to express human therapeutic molecules [107]. In all these studies, oral delivery of the engineered *L. lactis* was well tolerated with no side effects and no systemic exposure of the engineered bacteria. While safe, early efficacy was demonstrated in the context of Type 1 Diabetes, but not for IBD or oral mucositis. While the previous studies used human immune regulatory proteins, our group at Rise Therapeutics, in collaboration with Dr. D. Pascual at University of Florida, is further exploiting synthetic biology to reshape the host immune repertoire via microbiome-associated immune regulatory molecules. We have identified a protein called colonization factor antigen I (CFA/I), a fimbriae from enterotoxigenic *Escherichia coli*, that can engage dendritic cells lining the gastrointestinal tract to elicit Treg induction via the production of regulatory cytokines IL-10, IL-13, IL-35, and TGF-β [108–110]. The operon expressing CFA/I was integrated into the genome of a natural carrier probiotic to create R-2487. Recent preclinical studies demonstrated that R-2487 can induce Tregs, inhibit production of proinflammatory cytokines, and ameliorate clinical symptoms in the murine models of SS, as well as in murine models of arthritis, T1D, and multiple sclerosis [108,111–114]. In a murine model of SS, oral delivery of R-2487 significantly mitigated autoimmune disease severity by reducing the incidence of inflammatory infiltration into the submandibular and lacrimal glands and increasing Foxp3^+^, IL-10- and TGF-β-producing Tregs [114]. These studies demonstrated that CFA/I protein induced Tregs, which are key to controlling inflammation by reducing T effector cells [108,109,113]. Further, adoptive transfer of CFA/I-induced Tregs into mice with autoimmune disease conferred complete protection [108,109]. Thus, we believe that the mucosal induction of autoantigen Tregs by R-2487 represent an exciting, important, and safe new strategy for SS treatment. Moreover, our work supports the application of synthetic biology and development of engineered bacteria to specifically modulate the host immune repertoire. While pre-clinical data using engineered bacteria are very promising and intriguing, the clinical translation has lagged with a few clinical trials that ended for futility. Validation of this strategy and the targeted pathways in human clinical trials is needed to transform synthetic biology for microbiome from a promising strategy to reality.

## ADDITIONAL MICROBIOME-BASE INTERVENTIONS

The gut microbiome of SS patients presents a high relative richness of pathobionts [57], which are linked to the disease severity. A novel approach to modulate the gut microbiome is by using bacteriophages, which are viruses that specifically target and kill bacteria [118]. While their utilization has been focusing primarily to replace antibiotics, one can envision the use of bacteriophages to eradicate the pathobionts in SS patients with the goal of improving dysbiosis and modulating the immune response. Some preliminary data supporting this approach have been generated for gastrointestinal diseases [119]. However, only one paper reports the use of phages in a murine model of arthritis [120], hence much more work is needed to understand whether this strategy could be added to the therapeutic armamentarium.

The altered microbiome in SS patients may also activate the immune system via molecular mimicry and metabolite changes, leading to chronic inflammation and damage to the exocrine glands [46,121]. Gut metabolites alterations are also observed in other autoimmune diseases like systemic lupus erythematosus and rheumatoid arthritis [122–124]. These findings can be used to discover novel metabolites, in addition to the classical short chain fatty acids [125,126], that can be used as therapeutic molecules. Given the paucity of data so far, a long road ahead awaits scientists to prove that this strategy could be beneficial in SS.

## CONCLUSIONS

Understanding the cross-talk between the microbes inhabiting our body with the host is unveiling novel drug approaches that can lead to ground-breaking, efficacious, and safe new therapies. Novel, cutting-edge microbiome-based approaches with unique functional activity have the potential to be utilized as therapeutics, particularly in SS, a multifactorial disorder where classical pharmacological interventions may not be sufficient. Furthermore, by ‘hacking’ the microbiome genetic code and understanding how the microbiome controls the host immune system, we can also develop functionally-directed microbiome therapeutics, such as synthetic biology engineered bacteria, as precision targeted microbiome products. The initial hope for the gut microbiome as new therapeutic source is becoming a reality with a large number of clinical trials currently ongoing. These new microbiome therapeutic approaches, particularly in the context of regulating the immune repertoire of the host, have tremendous potential for application in the treatment of SS.

## Figures and Tables

**Table 1.** Human studies using microbiome interventions.

Conditions	Intervention	Results	ref

immune-mediated dry eye (DE)	FMT	Safety confirmed Partial disease improvement observed	[53]
dry eye	Prebiotics and probiotics	Improved ocular surface disease index	[67]
SS	Probiotics	Reduction of the candida load but no significant changes compared to placebo	[68]
SS	Probiotics	No beneficial effects noted	[69]

**Table 2.** Clinical trials with engineered *L. lactis.*

Condition	Intervention	Phase	Results	ref

Crohn’s disease	Engineered *L. lactis* expressing IL-10	1	Safety was confirmed	[87]
Ulcerative mucositis	*L. lactis* expressing human trefoil factor 1 (hTFF1)	1b	Safety was confirmed	[88]
Ulcerative colitis	Engineered *L. lactis* expressing IL-10	2	Primary endpoint not met	[115]
Ulcerative mucositis	*L. lactis* expressing human trefoil factor 1 (hTFF1)	1b	Safety was confirmed	[88]
Ulcerative mucositis	*L. lactis* expressing human trefoil factor 1 (hTFF1)	2	Trial terminated due to lack of efficacy	[116]
Type 1 Diabetes	engineered *L. lactis* strain expressing proinsulin and IL-10	1b	Safety was confirmed Improved C-peptide stabilization	[117]

## Data Availability

Data sharing not applicable to this article as no datasets were generated or analyzed during the current study.
